# Efficacy of gabapentin versus trospium chloride for prevention of catheter-related bladder discomfort inside the surgical intensive care unit: a prospective, randomised, controlled clinical study

**DOI:** 10.62838/jccm-2026-0018

**Published:** 2026-07-27

**Authors:** Ahmed Moustafa Mohamed, Wessam Zaher Selima

**Affiliations:** Department of Anesthesia, Intensive Care and Pain Management, Faculty of Medicine, Ain Shams University, Cairo, Egypt

**Keywords:** catheter-related bladder discomfort, CRBD four-point severity scale, gabapentin, surgical intensive care unit, trospium chloride

## Abstract

**Introduction:**

Catheter-related bladder discomfort (CRBD) after perioperative catheterisation of the urinary bladder (COUB) is not uncommon.

**Aim of the study:**

We evaluated the efficacy of both oral gabapentin and trospium in preventing CRBD during the early postoperative period in patients admitted to the surgical intensive care unit (S-ICU).

**Material and Methods:**

120 patients aged 20–65 years, ASA I, II or III who were admitted to S-ICU after undergoing elective spinal surgery (ESS) with COUB were included. They were randomly assigned to be administered either an oral 400 mg gabapentin capsule (Group G) or an oral 60 mg slow-release trospium chloride capsule (Group T) or nothing (Group C). The primary goal was the occurrence of CRBD and its severity at 1, 2, 6, 12, and 24 hours after the study drug administration (SDA).

**Results:**

Group G and group T had a statistically significant lower incidence of CRBD than group C at 1, 2, 6, 12, and 24 hours after SDA, respectively. Both had considerably lower severity than group C in the first two hours only (P= 0.001 and 0.001, respectively). Group T had non-significantly lower incidence and severity of CRBD than group G. Group G had significantly lower mean total fentanyl requirements for up to 24 hours after SDA than group T and group C (P < 0.001).

**Conclusion:**

Both oral gabapentin capsules and slow release trospium chloride capsules administered postoperatively, significantly decreased both the incidence of CRBD and its severity in the early postoperative period amongst S-ICU patients, without significant differences between the two drugs.

## Introduction

Patients with catheter-related bladder discomfort (CRBD) complain of symptoms almost identical to those of overactive bladder syndrome (OABS) such as; painful urinary irritation and burning in suprapubic and pelvic areas, frequency and/or urgency [1]. It can trigger serious behavioural reactions such as agitation, restlessness, delirium or even traumatic self removal of the catheter. Systemic responses such as hypertension and dysrhythmia can also occur with negative psychological impacts [2].

The main pathophysiology of CRBD and OABS is catheter-induced irritation of the bladder acetylcholine release, which stimulates urothelial M2 and M3 muscarinic receptors, leading to involuntary detrusor muscle contractions [3].

Antimuscarinic drugs including trospium chloride, darifenacin, solifenacin, tolterodine and oxybutynin are the first choice drugs for OABS, so they have been studied for the prevention of perioperative CRDB with variable degrees of success [4,5]. Trospium chloride drug is a non selective antimuscarinic agent that is effective in managing resistant cases of OABS [6]. Extended-release trospium chloride is also effective with the merit of a single daily dose [7].

Some antiepileptic drugs, such as pregabalin and gabapentin, have been also reported to be effective in managing resistant cases of OABS through the inhibition of up regulated bladder afferent C-fibres [8]. Therefore, both are used for prevention of CRDB, with subsidiary analgesic effect [9,10]. Additionally, other drugs are used in CRDB prevention such as ketamine, tramadol, paracetamol and dexmedetomidine [11,12,13,14]. We aimed to evaluate the efficacy of both gabapentin and trospium chloride in preventing CRBD during early postoperative period in patients who admitted to surgical intensive care unit (S-ICU) for postoperative care (POC) after undergoing elective spinal surgery (ESS) and requiring intraoperative catheterisation of the urinary bladder (COUB).

## Materials and methods

### Ethical approval, study design, and participants

This was a prospective, randomised, double-blind, and controlled clinical trial study that received ethical approval from the research ethics committee (REC) of the faculty of medicine, Ain Shams University in Cairo, Egypt (Approval code: FWA000017585-FMASU R37/2024, 02-22-2024) and was registered at ClinicalTrials.gov with ID: NCT06346522 [https://clinical-trials.gov/study/NCT06346522], 03-29-2024. Before starting the study, all participants consented both verbally and in written form.

It was conducted on 120 patients in the S-ICUs of Ain Shams University Hospitals in Cairo, Egypt, from April 10 to September 20, 2024. Inclusion criteria were patients aged 20–65 years, American Society of Anaesthesiologists (ASA) physical status class of I, II or III, who were admitted to the S-ICU after undergoing ESS and requiring COUB. Patients who already had developed CRBD symptoms or with urinary bladder pathology such as OABS or neurological bladder, allergies to the study drugs, moderate to severe hepatic disease, renal impairment with a creatinine clearance (CrCl) <30 ml.min^−1^, NPO (nil per os), severe chronic constipation or ileus, or narrow angle glaucoma were excluded.

### Randomisation and blinding procedures

The S-ICU specialist sent a request for randomisation to an administrator once a patient met the eligibility criteria for enrolment, and then the administrator sent an opaque sealed envelope delivered to the intensivist in charge containing the patient assignment to the groups. Randomisation was done by using simple random (probability) sampling using random number tables generated via a computer. The consented and enroled 120 patients were randomly allocated into three parallel arms and equivalent (1:1:1 ratio) groups, either group G (40 patients), group T (40 patients), or group C (40 patients). Blinding included the patients, the outcome assessors, and the investigators. The oral study drugs capsules were packed in small code-labelled plastic bags with a zip lock. It was only after the completion of the study that the codes were then broken for analysis. The study end point was 24 hours after SDA.

### Study procedures and interventions

After satisfactory pain control and full recovery in the post-anesthesia care unit, the patient was transferred and admitted to S-ICU as a case of POC after ESS. Admission was for medical, surgical or anaesthesia-related causes.

Standard monitoring via GE Healthcare monitors (Careescape^TM^ B650, General Electric, Boston, Mass, USA) with standard postoperative clinical and laboratory follow-up.

After resumption of oral feeding according to certain criteria [15], and assessment for the absence of CRBD symptoms by the S-ICU specialist, the oral study drug was administered with sips of water within half an hour of admission. Enroled participants were randomly assigned to either the group G who administered oral gabapentin 400 mg capsule (Gaptin^R^, Delta Pharma Company, Cairo Egypt), the group T who administered oral slow-release trospium chloride 60 mg capsule (Trospikan^R^ SR, Hikma Pharma Company, Cairo, Egypt) as a single daily dose, or group C who administered nothing (Control group; non-pharmacological standards of care group).

The incidence and severity of CRBD were assessed by the S-ICU specialist using the CRBD four-point severity scale after the study drug administration (SDA). Assessment of postoperative pain (POP) severity and control was performed by the S-ICU specialist using the visual analogue pain scale (VAS; 0–10). Slow IV 25 µg fentanyl was administered as an analgesic for POP when VAS score >3 and as a rescue drug for moderate or severe CRBD, and another slow IV 25 µg fentanyl was re-administered if no improvement within 10 minutes, with measuring of the total fentanyl requirements in the first 24 hours in micrograms (µg in 24 hours) after SDA. Side effects of the used drugs were recorded and managed accordingly. Assessment of the sedation level one hour after SDA was performed using the Ramsay sedation scale (RSS). Patients continued their monitored care in the S-ICU until proper stabilisation and management of the causes of their S-ICU admission.

### Data collection and recording

Patient demographic data, causes of POC in the S-ICU, and spine surgery site and number (%) for each cervical, dorsal, or lumbar were recorded.

### Outcome measures

*Primary outcome:* It was the incidence (yes/no) and severity of CRBD at 1, 2, 6, 12, and 24 hours after SDA. Severity was assessed and recorded by the CRBD four-point severity scale developed by Agarwal et al.[16] as follows:

No CRBD (scale 0): No urinary urgency or suprapubic discomfort; mild CRBD (scale 1): informed by the patient on enquiring only; moderate CRBD (scale 2): informed by the patient without being enquired; severe CRBD (scale 3): informed by the patient himself and associated with behavioural changes such as attempts to catheter removal, agitation, or restless movements.

*Secondary outcomes:* They were total fentanyl requirements in the first 24 hours after SDA in micrograms; VAS was assessed at 1, 2, 6, 12, and 24 hours after SDA; RSS was assessed after one hour of SDA; and the side effects of the used drugs. The study end point was 24 hours after SDA.

### Sample Size

It was calculated by using the power analysis and sample size software programme (PASS 15, NCSS, USA), setting the power at 80%, the alpha error at 5%, and reviewing the results from previous studies [17, 18] revealed that using gabapentin decreased the incidence of CRBD to 50% in contrast to 80% in the placebo group, and using trospium decreased the incidence of CRBD to 22% compared with 66% in the placebo group. Assuming a medium effect size difference between the groups concerning the incidence of CRBD (d=0.3) and after 10% adjustment for the dropout rate, a sample of 40 patients for each group would be needed.

### Statistical Analysis

It was performed using the Statistical Package for Social Science (SPSS) version 27 (IBM Corp, Armonk, NY, USA) after data were collected, revised and coded. The quantitative parametric data are presented as the mean, standard deviations and ranges. The quantitative non-parametric data are presented as the median and interquartile ranges (1Q, 3Q). The qualitative variables are presented as numbers and percentages and were compared utilising the Chi-square test and/or Fisher’s exact test as soon as the expected count in any cell was < 5. One way ANOVA (Analysis of Variance) test, was utilised for comparisons between more than two independent groups with quantitative data and parametric distributions, whereas the Kruskall-Wallis test was utilised for comparisons with non-parametric distributions. The confidence interval was set to 95%, while the margin of error was set to 5%. Accordingly, the P value was considered significant when P <0.05.

## Results

**Fig. 1.**
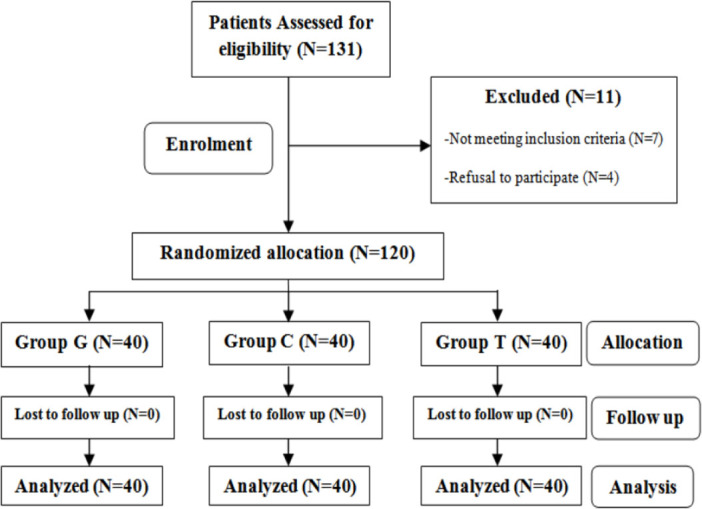
Study flowchart in accordance with CONSORT 2025. G: gabapentin, C: control, T: trospium, CONSORT: consolidated standards of reporting trials.

One hundred and thirty-one patients were assessed for eligibility, with an enrolment of 120 who completed the whole study (Figure 1).

There were no statistically significant differences regarding the patients' demographic data and clinical characteristics between group G, group T and group C (Table 1).

**Table 1.** Patients' demographic data and clinical characteristics

**Variables**		**Group G (N=40)**	**Group T (N=40)**	**Group C (N=40)**	**Used test value**	**P value**
Age in years	M ± SD	51.98 ± 6.59	51.47 ± 8.29	50.38 ± 8.98	0.413^*^	0.663
	Range	40 – 64	40 – 65	25.7 – 69.81		

Males/Females		31/9	32/8	30/10	0.287^†^	0.866

BMI (kg.m^−2^)	M ± SD	25.2 ± 3.76	26.82 ± 3.8	26.13 ± 4.01	1.789^†^	0.172
	Range	20 – 32	20 – 32	16.44 – 34.31		

ASA classification	I	16 (40.0%)	17 (42.5%)	18 (45.0%)	0.569^†^	0.966
	II	18 (45.0%)	16 (40.0%)	17 (42.5%)		
	III	6 (15.0%)	7 (17.5%)	5 (12.5%)		

Values are presented as mean ± SD, range, and numbers (%). G: gabapentin, T: trospium, C: control, BMI: body mass index, Kg: kilogramme, M: metre, ASA: American Society of Anaesthesiologists (ASA).

*: One-way ANOVA test;

†: Chi-square test.

With respect to medical, surgical, and anaesthesia-related causes of postoperative S-ICU admission, there is no considerable difference between the three groups, with the medical causes being the main causes of POC in the S-ICU (Table 2). Additionally, there is no significant difference concerning the anatomical sites of spine surgeries, with the lumbar vertebral surgeries being the main sites in the three groups (Table 2).

**Table 2.** Causes of postoperative care in the intensive care unit and sites of spine surgery and their number (%)

**Causes of POC in the S-ICU**	**Group G (N=40)**	**Group T (N=40)**	**Group C (N=40)**	**Used test value**	**P value**
Medical causes	34 (85.0%)	36 (90.0%)	35 (87.5%)	0.739*	0.946
Surgical causes	4 (10.0%)	3 (7.5%)	4 (10.0%)		
Anaesthesia-related causes	2 (5.0%)	1 (2.5%)	1 (2.5%)		

Spine surgery site					
Cervical	7 (17.5%)	6 (15%)	5 (12.5%)	1.583*	0.812
Dorsal	3 (7.5%)	2 (5%)	1 (2.5%)		
Lumbar	30 (75%)	32 (80%)	34 (85.0%)		

Values are presented as numbers (%). G: gabapentin, T: trospium, C: control, POC: postoperative care, S-ICU: surgical intensive care unit.

*: Chi-square test.

**Fig. 2.**
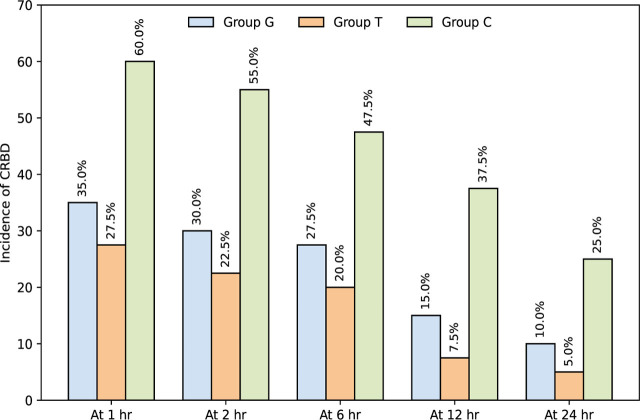
The incidence of CRBD at 1, 2, 6, 12, and 24 hours after the study drugs administration. CRBD: catheter-related bladder discomfort, G: gabapentin, T: trospium, C: control.

**Fig. 3.**
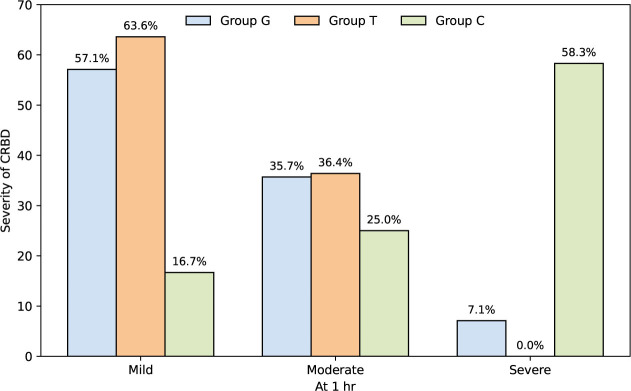
The severity of CRBD at 1 hour after the study drugs administration. CRBD: catheter-related bladder discomfort, G: gabapentin, T: trospium, C: control.

**Fig. 4.**
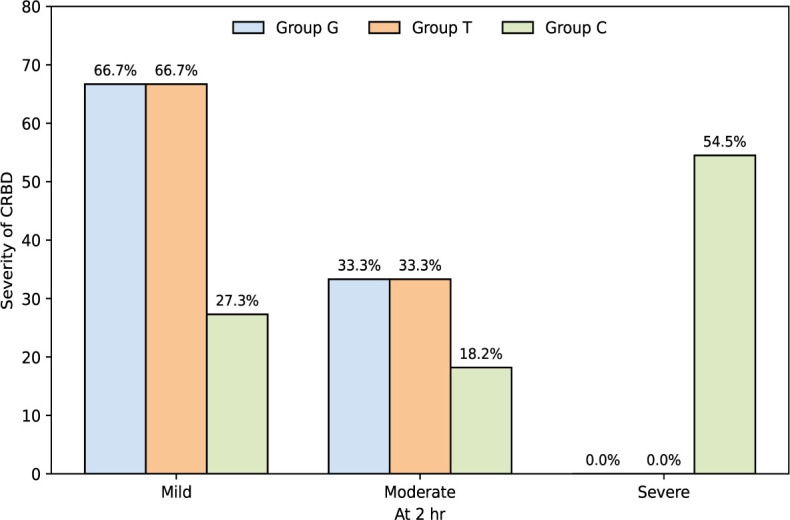
The severity of CRBD at 2 hours after the study drugs administration. CRBD: catheter-related bladder discomfort, G: gabapentin, T: trospium, C: control.

Group C had a statistically significant higher incidence of CRBD (60%, 55%, 47.5%, 37.5%, and 25%) than group G (35%, 30%, 27.5%, 15%, and 10%) and group T (27.5%, 22.5%, 20%, 7.5%, and 5%) at 1, 2, 6, 12, and 24 hours after SDA, respectively (Table 3, Figure 2). Additionally, the severity of CRBD at 1, 2, 6, 12, and 24 hours after the SDA was higher in group C than in group G and group T; however, this difference was significant only in the first two hours (P= 0.001 and 0.001, respectively), (Figure 3 and 4). Severity was in-significantly lower in group T than in group G, with the occurrence of severe CRBD once in the first hour after SDA in group G (Table 3).

**Table 3.** Incidence and severity of catheter-related bladder discomfort at 1, 2, 6, 12, and 24 hours after intake of the study drugs

**CRBD incidence in hours**		**Group G (N=40)**	**Group T (N=40)**	**Group C (N=40)**	**Used test value**	**P value**
At 1 hour		14 (35.0%)	11 (27.5%)	24 (60.0%)	9.589^*^	0.008^†^
At 2 hours		12 (30.0%)	9 (22.5%)	22 (55.0%)	10.076^*^	0.006^†^
At 6 hours		11 (27.5%)	8 (20.0%)	19 (47.5%)	7.471^*^	0.024^†^
At 12 hours		6 (15.0%)	3 (7.5%)	15 (37.5%)	12.188^*^	0.002^†^
At 24 hours		4 (10.0%)	2 (5.0%)	10 (25.0%)	7.500^*^	0.024^†^

**CRBD severity in hours**						

At 1 hour	Mild	8 (57.1%)	7 (63.6%)	4 (16.7%)	18.393^*^	0.001^†^
	Moderate	5 (35.7%)	4 (36.4%)	6 (25.0%)		
	Severe	1 (7.1%)	0 (0.0%)	14 (58.3%)		

At 2 hours	Mild	8 (66.7%)	6 (66.7%)	6 (27.3%)	16.004^*^	0.003^†^
	Moderate	4 (33.3%)	3 (33.3%)	4 (18.2%)		
	Severe	0 (0.0%)	0 (0.0%)	12 (54.5%)		

At 6 hours	Mild	8 (72.7%)	6 (75.0%)	7 (36.8%)	9.347^*^	0.053
	Moderate	3 (27.3%)	2 (25.0%)	5 (26.3%)		
	Severe	0 (0.0%)	0 (0.0%)	7 (36.8%)		

At 12 hours	Mild	4 (66.7%)	2 (66.7%)	8 (53.3%)	1.371^*^	0.849
	Moderate	2 (33.3%)	1 (33.3%)	5 (33.3%)		
	Severe	0 (0.0%)	0 (0.0%)	2 (13.3%)		

At 24 hours	Mild	3 (75.0%)	2 (100.0%)	7 (70.0%)	1.267^*^	0.867
	Moderate	1 (25.0%)	0 (0.0%)	2 (20.0%)		
	Severe	0 (0.0%)	0 (0.0%)	1 (10.0%)		

Values are presented as numbers (%). G: gabapentin, T: trospium, C: control, CRBD: catheter-related bladder discomfort.

*: Chi-square test;

†: P < 0.05, indicating statistical significance.

**Fig. 5.**
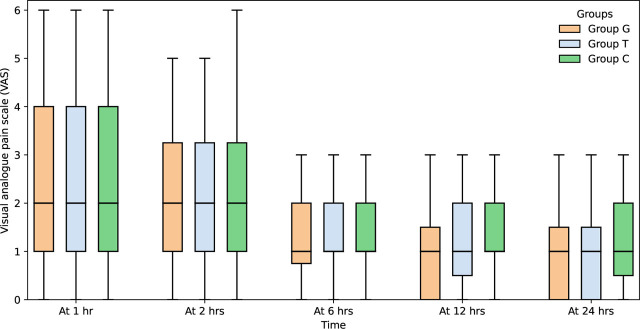
Visual analogue pain scale (VAS) at 1, 2, 6, 12, and 24 hours after the study drugs administration. G: gabapentin, T: trospium, C: control.

**Fig. 6.**
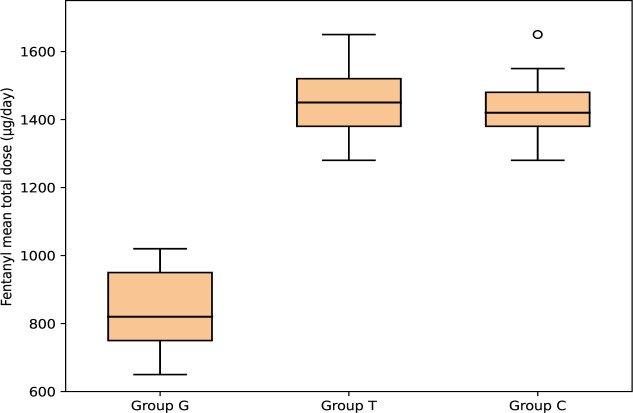
Fentanyl total requirements in the first 24 hours after the study drugs administration in micrograms (µg) in 24 hours. G: gabapentin, T: trospium, C: control.

The visual analogue pain scale (VAS) was comparable amongst the three groups with an overall low median VAS. However, group G had statistically significantly lower VAS at 6 and 12 hours after SDA than group T and group C (P = 0.003 and 0.014, respectively), (Table 4 and Figure 5). Additionally, group G had significantly lower mean total fentanyl requirements in the initial 24 hours after SDA (850.07 ± 122.63 µg) than group T (1437.02 ± 103.67 µg) and group C (1425.58 ± 88.88 µg), (P < 0.001), (Table 4 and Figure 6). Finally, the side effects of the utilised drugs, including RSS, were minimal amongst the three groups without substantial difference, with an overall low mean RSS in groups G, T and C (2.38 ± 0.67, 2.23 ± 0.73, 2.08 ± 0.69, respectively), (Table 5).

**Table 4.** Visual analogue pain scale (VAS) at 1, 2, 6, 12, and 24 hours after the study drug administration and the total fentanyl requirements (µg) in 24 hours

**Timed VAS**		**Group G (N=40)**	**Group T (N=40)**	**Group C (N=40)**	**Used test value**	**P value**
At 1 hour	Median	2 (1 – 4)	2 (1 – 4)	2 (1 – 4)	0.123*	0.941
	Range	0 – 6	0 – 6	0 – 6		
At 2 hours	Median	2 (1 – 3)	2 (1 – 4)	2 (1 – 4)	0.212*	0.900
	Range	0 – 5	0 – 5	0 – 6		
At 6 hours	Median	1 (0 – 2)†	2 (1 – 2)‡	2 (1 – 2)‡	11.489*	0.003§
	Range	0 – 3	0 – 3	0 – 3		
At 12 hours	Median	1 (0 – 1)†	2 (0 – 2) ‡	2 (1 – 2)‡	8.536*	0.014§
	Range	0 – 3	0 – 3	0 – 3		
At 24 hours	Median	1 (0 – 1)	2 (0 – 2)	2 (0 – 2)	5.560*	0.062
	Range	0 – 3	0 – 3	0 – 3		

Total fentanyl doses (µg)						
Mean ± SD		850.08 ± 122.63†	1437.03 ± 103.67‡	1425.58 ± 88.88‡	401.281¶	< 0.001§
Range		654 – 1025	1275 – 1661	1275 – 1661		

Values are presented as median (1Q, 3Q), range, and mean ± SD. VAS: visual analogue pain scale. G: gabapentin, T: trospium, C: control, µg: micrograms.

*: Kruskall-Wallis test;

† and

‡: indicate significant difference between groups;

§: P < 0.05, indicating statistical significance;

¶: One-way ANOVA test.

**Table 5.** Side effects of the study drugs

**Drugs’ side effects**		**Group G (N=40)**	**Group T (N=40)**	**Group C (N=40)**	**Used test value**	**P value**
Nausea/vomiting		1 (2.5%)	0 (0.0%)	0 (0.0%)	2.017*	0.365
Facial flushing		0 (0.0%)	1 (2.5%)	0 (0.0%)	2.017*	0.365
Dry mouth		2 (5.0%)	4 (10.0%)	1 (2.5%)	2.124*	0.346
Headache		0 (0.0%)	1 (2.5%)	0 (0.0%)	2.017*	0.365
Light headiness		1 (2.5%)	1 (2.5%)	0 (0.0%)	1.017*	0.601
Constipation		1 (2.5%)	3 (7.5%)	1 (2.5%)	1.670*	0.434
Agitation		0 (0.0%)	0 (0.0%)	1 (2.5%)	2.017*	0.365
Ramsay sedation score	M ± SD	2.38 ± 0.67	2.23 ± 0.73	2.08 ± 0.69	1.843†	0.163
	Range	1 – 3	1 – 3	1 – 3		

Values are presented as numbers (%), mean ± SD, range. G: gabapentin, T: trospium, C: control.

*: Chi-square test;

†: One-way ANOVA test.

## Discussion

This single-centre, randomised, controlled trial illustrated that both the oral 400 mg gabapentin capsule and the slow-release trospium chloride 60 mg capsule administered postoperatively, after resumption of oral feeding, significantly reduced both the incidence and severity of CRBD in the early postoperative period (POP), amongst patients admitted to the S-ICU after undergoing ESS and who required COUB. Uniquely, gabapentin considerably reduced mean total fentanyl requirements.

Agreeing with our study results but with different intervention protocols, Agarwal et al. [17] also reported that the administration of 600 mg gabapentin orally one hour before induction of anaesthesia in patients who underwent percutaneous nephrolithotomy (PCNL), reduced the occurrence of postoperative CRBD (50%, 61%, 55%, and 37%) and its severity at 0, 1, 2 and 6 hours, respectively. It also significantly reduced the postoperative pain and the total fentanyl consumption.

In studies with similar methodology to Agarwal et al. [17] but in patients who underwent ESS, administration of oral 150 mg pregabalin [10] or 60 mg trospium chloride (ER) [18], markedly reduced the incidence of postoperative CRBD (36.6%, 36.6%, 30%, 16.6 % versus 22%, 28%, 22%, and 9%) and its severity at 0, 1, 2 and 6 hours, respectively. Pregabalin also led to a significant reduction in postoperative fentanyl consumption, but not trospium chloride.

Comparably, a study [16] reported that oral oxybutynin and tolterodine decreased the incidence of CRBD by 23–25 %, respectively. Also, Tauzin et al. [19] demonstrated a decrease in the incidence of CRBD by 48% after radical prostatectomy using oxybutynin sublingually. However, a mixed drug effect was obtained, as 600 mg oral gabapentin was used as a premedication and 100 mg IV tramadol was used during wound closure. Both of these drugs also lower the occurrence of CRBD.

We selected spinal surgery for our study to avoid the confusion in differentiating the CRBD from pain or spasm associated with genitourinary surgery.

Moreover, we administered the study drugs to patients orally after their full anaesthesia recovery, transfer and admission to the S-ICU. This isolates the effect of drugs used in general anaesthesia which may influence the incidence of CRBD. Two studies [20,21] revealed that the use of IV glycopyrrolate just as a premedication or as a part of a reversal drug to antagonise neuro-muscular blockade decreased the incidence of CRBD. Additionally, another two studies [22,23] reported that inhalational sevoflurane decreased the incidence of immediate postoperative CRBD (up to 1 hour) compared with desflurane and propofol.

Furthermore, we administered slow release (SR) 60 mg trospium chloride, first for its long action and second because 60 mg is the most efficient once-daily dose for management of OABS [24]. Also, we administered 400 mg gabapentin, first for its long action and second because its initial doses range from 100 to 300 mg for most painful syndromes, such as post-herpetic neuralgia, neuropathic pain, fibromyalgia, and restless leg syndrome.

Conversely, Agrawal et al. [17] administered 600 mg gabapentin to patients who underwent elective PCNL, whereas Bala et al. [9] administered either 600 mg or 1200 mg gabapentin capsules to patients who underwent transurethral resection of bladder tumours. These larger doses may be needed because of the associated visceral pain and spasm with urological surgeries, which mimic CRBD.

In our study, both drugs were safe with minimal side effects and without significant differences between them. Dry mouth occurred in two patients (5%) in group G, four patients (10%) in group T and one patient (2.5%) in group C. Additionally, there was no excessive sedation, as a RSS of 2 may be advantageous rather than a side effect, as the patient is awake, oriented, cooperative, and tranquil and does not interfere with the CRBD assessment.

Previous study [16] revealed a greater incidence of dry mouth peaking at one hour postoperatively in the oxybutynin arm (58.9%) and in the tolterodine arm (55.1%). Srivastava et al. [10] also reported sedation by utilising oral pregabalin with a mean RSS of 2.63 ± 0.67.

This study has limitations. First, gabapentin was approved by the Food and Drug Administration (FDA) to manage certain forms of neuropathic pain, post-herpetic neuralgia, fibromyalgia, and partial seizures but not for treating urologic dysfunction compared to trospium chloride, which is used for management of OABS. However, gabapentin is utilised off-label for management of neurogenic detrusor overactivity [25] and refractory genitourinary tract pain [26]. Second, we used oral drugs that cannot be taken by patients with prolonged NPO (nil per os) status. Third, we used fixed doses for both drugs, so further studies with multiple lower doses are needed to evaluate the dose response titration and the minimal effective doses for preventing CRBD with the least side effects. Finally, this was a single-centre study of patients admitted to S-ICU. Further multicenter studies on more patients and on patients in different ICUs (e.g. medical, cardiac, or neurological ICUs) are needed for the generalisation of the results.

## Conclusion

Both oral gabapentin 400 mg capsule and slow release trospium chloride 60 mg capsule administered postoperatively markedly decreased the incidence of CRBD and its severity in the early postoperative period amongst patients admitted to the S-ICU for POC after undergoing ESS and requiring COUB, compared to non-pharmacological standard of care after COUB, without a significant difference between the two drugs, and with minimal side effects. Uniquely, gabapentin significantly reduced the total fentanyl consumption compared to trospium and non-pharmacological standards of care after COUB.
